# Solitary Fibrous Tumor of the Right Elbow: A Case Report

**DOI:** 10.7759/cureus.88175

**Published:** 2025-07-17

**Authors:** Maciej Mach, Tomasz Ostrowski, Karol Maciejewski, Rafal Ostrowski, Zbigniew Gałązka

**Affiliations:** 1 Department of General, Vascular, Endocrine and Transplant Surgery, Medical University of Warsaw, Warsaw, POL

**Keywords:** elbow joint, extrapleural sft, mesenchymal neoplasm, soft-tissue tumor, solitary fibrous tumor (sft)

## Abstract

We report a case of a 58-year-old male patient with an extrapleural solitary fibrous tumor (SFT) arising in the soft tissues surrounding the right elbow joint, which is a highly unusual anatomical location. The lesion exhibited slow, progressive growth over the years and was associated with intermittent neurological symptoms. Surgical excision was performed due to tumor growth and progressive neurological deficits to relieve discomfort and prevent further neural damage. Imaging demonstrated a well-circumscribed soft tissue mass, and complete surgical excision was undertaken. Histopathological evaluation revealed a well-circumscribed, encapsulated tumor measuring 5×4.8×3.5 cm with fibrous areas. Immunohistochemical staining showed strong positivity for Cluster of Differentiation (CD)34 and CD99, consistent with the diagnosis of SFT. This case highlights the necessity of including SFT in the differential diagnosis of slow-growing soft tissue masses in various, often atypical locations. Taking into account the potential for local recurrence and malignant transformation, long-term surveillance after resection is recommended, even in histologically benign cases.

## Introduction

A solitary fibrous tumor (SFT) is a rare mesenchymal fibroblastic neoplasm that was first described in the pleura by Klemperer and Rabin in 1931 [1]. Although SFTs were initially believed to arise solely from the pleura, they are now known to occur in virtually any anatomical location, including the central nervous system, retroperitoneum, head and neck, and extremities [2,3]. The clinical behavior of these tumors ranges from indolent to highly aggressive, and their histologic appearance may not always correlate with clinical outcomes [4].

The incidence of SFTs is estimated at approximately one per million individuals annually, affecting men and women equally, with a typical age of presentation between 50 and 60 years [5]. Despite advances in understanding their molecular and histological characteristics, their etiology remains largely unknown, and no specific environmental or genetic risk factors have been definitively identified [3].

Clinically, SFTs are often asymptomatic and discovered incidentally. However, depending on the location, they can exert mass effect or invade nearby structures, leading to symptoms such as pain, functional impairment, or hypoglycemia due to ectopic Insulin-like growth factor (IGF)-2 production (Doege-Potter syndrome) [2,3].

SFTs in the extremities are particularly rare, accounting for a small fraction of cases reported in the literature [6]. Their occurrence in anatomically complex and confined spaces such as the elbow joint presents unique diagnostic and surgical challenges. These include difficulties in imaging interpretation, risks related to the tumor’s vascularity, and the need to balance complete tumor excision with the preservation of joint function and neurovascular structures [7]. Because of these complexities, the management of extremity SFTs requires a multidisciplinary approach and careful preoperative planning.

This case report is clinically significant as it details the rare presentation of an SFT localized near the right elbow joint, highlighting the diagnostic difficulties and surgical considerations inherent to this location. By sharing the clinical course, imaging findings, surgical strategy, and long-term outcomes, we aim to contribute valuable insight to the limited existing literature on extremity SFTs, ultimately enhancing awareness and guiding management of similar cases in the future [8].

## Case presentation

In January 2011, a 58-year-old male patient was admitted to our clinic for the scheduled surgery of a soft tissue tumor in the area of the right elbow joint. The tumor was first noticed approximately 20 years ago, shortly after a vessel cannulation during hospitalization; however, no causal relationship between the procedure and tumor development can be established, and this temporal association may be coincidental. The patient had been experiencing intermittent numbness in the fingers that worsened over the past three years. His prior medical history revealed cholecystectomy, two cataract surgeries, trigeminal neuritis, and hypertension. On general examination, his blood pressure was 140/75 mmHg. His abdomen was soft and painless, and there was no peripheral edema. There was a visible tumor in the area of the right elbow joint. Due to the enlarging tumor and worsening neurological symptoms, a decision was made to perform surgical excision of the lesion to alleviate symptoms and prevent further damage to the surrounding neural structures.

**Figure 1:**
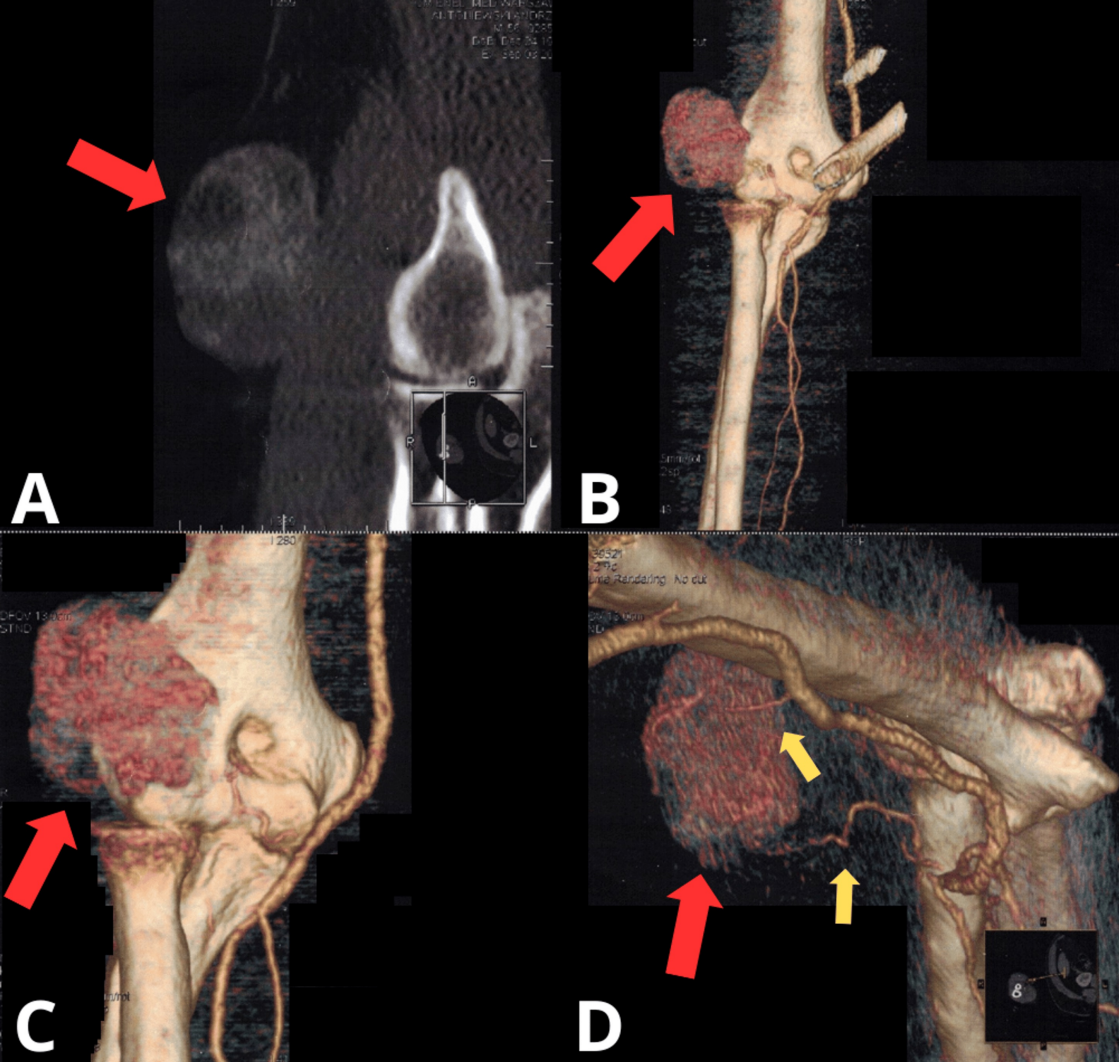
Imaging diagnostics of the tumor mass on CT CT: Computed Tomography; 2D: Two-dimensional; 3D: Three-dimensional (A) Axial 2D CT view showing the tumor mass; (B and C) 3D reconstruction of the elbow joint, anterior projection; (D) 3D reconstruction of the elbow joint, lateral projection. Red arrow indicates the tumor mass and the yellow arrow indicates the feeding vessels supplying the tumor.

Computed tomography (CT) angiography (Figure 1) revealed a very richly vascularized soft tissue tumor in the area of the right elbow joint, with the main blood supply coming from two branches: from the brachial artery at the level of the elbow joint gap and the radial artery at the level of the radial head.

**Figure 2:**
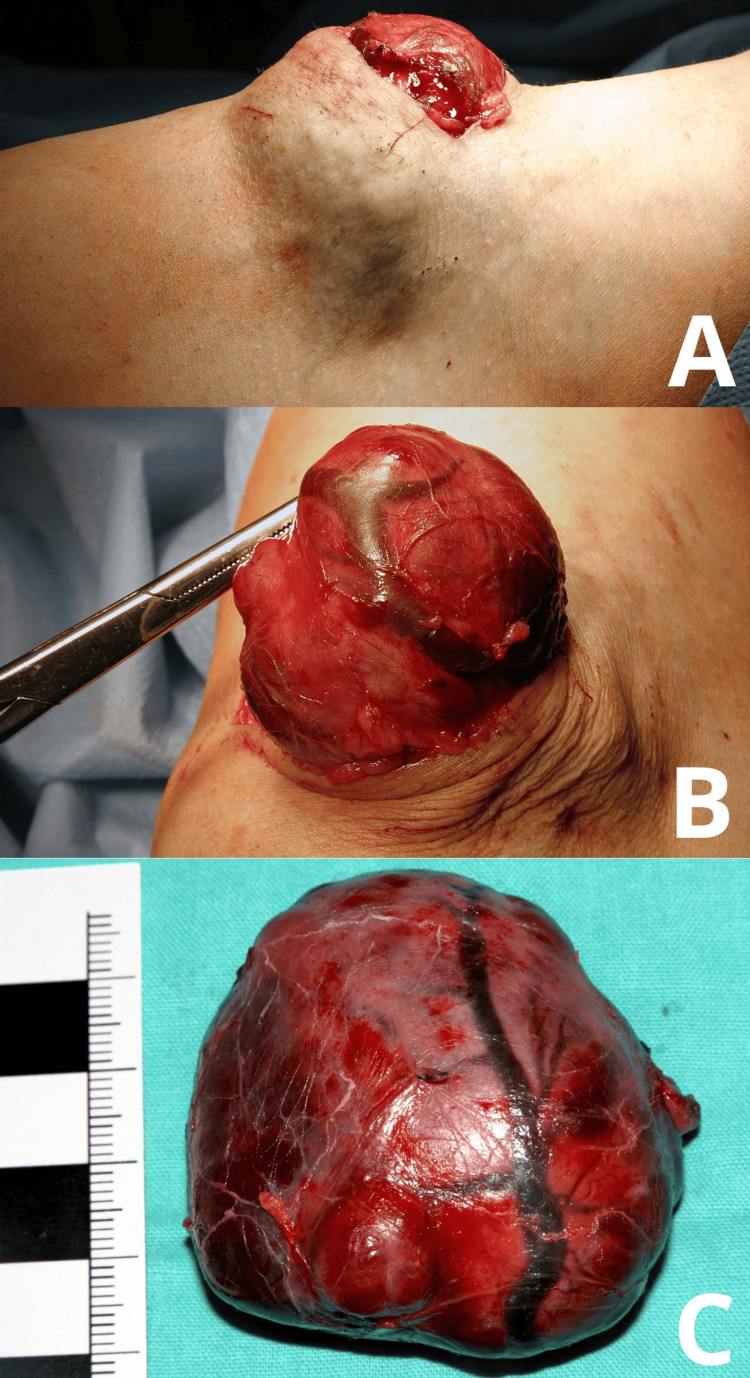
Intraoperative photographs (A) Visible tumor mass following the initial skin incision; (B) Tumor after dissection, with a visible feeding vessel branch; (C) Excised tumor mass.

Surgery was performed on Jan 17, 2011. An incision was made in the area of the right cubital fossa, reaching the large, highly vascularized tumor (Figure 2A).

The tumor was surrounded by a strong fibrous capsule on which large venous vessels were visible. Some of these vessels were cut and ligated, while the remaining vessels were retracted to the medial side of the cubital fossa (Figure 2B). The tumor was dissected by ligating the visible arterial vessels supplying it, and was completely removed (Figure 2C). A redon drain was left in place in the operated area. The course of the surgery was without complications. The patient was discharged home in good condition.

**Figure 3:**
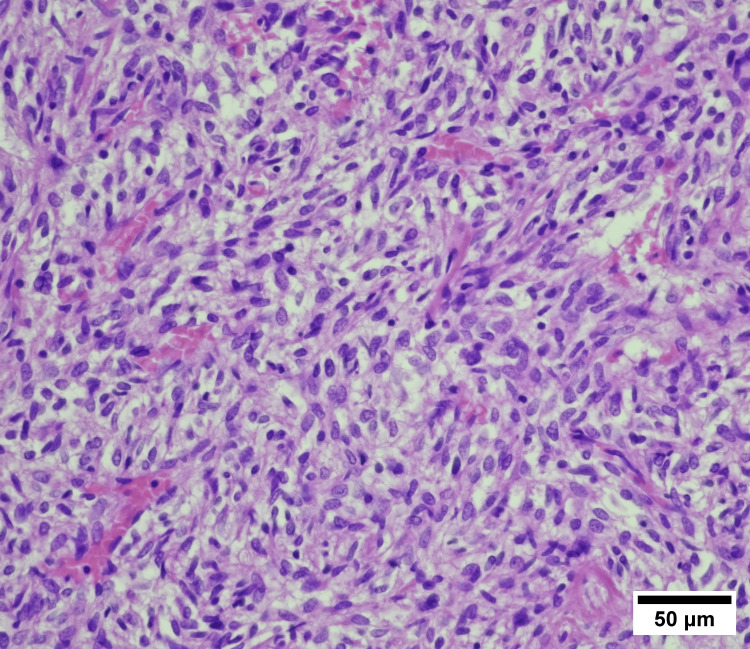
Microscopic image of a histopathological specimen from the tumor

Histopathological diagnosis revealed that the resected tumor was encapsulated, measuring 5x4.8x3.5 cm. On sectioning, the tumor was relatively firm, beige-brown in color, and likely with areas of fibrosis. Macroscopically, the tumor appeared to be completely enucleated, and microscopic examination revealed a SFT. Immunohistochemistry showed that it was positive for Cluster of DIfferentiation (CD)34 and CD99 (Figure 3).

Two follow-up ultrasound examinations of the right upper extremity were conducted in 2014 and 2020. No pathological tissue echoes or fluid collections were observed in the area of the surgical scar. Arteries and veins in the cubital fossa were patent.

**Figure 4:**
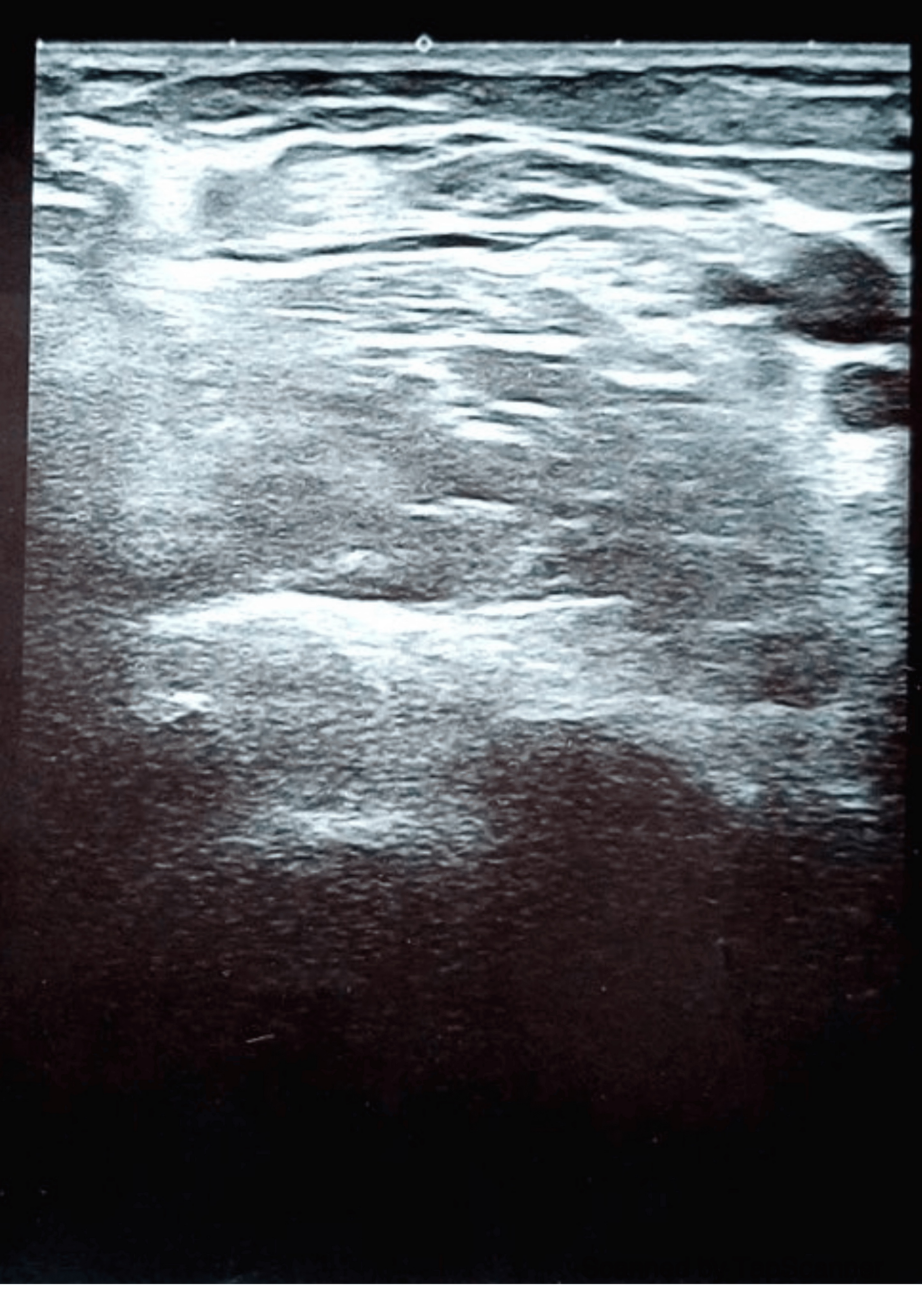
Ultrasound image of the tumor bed 14 years after the excision

Ultrasound imaging performed in 2025 demonstrated no evidence of residual or recurrent tumor at the surgical site (Figure 4).

The examination revealed normal tissue architecture with no abnormal mass, hypoechoic lesion, or vascular irregularity within the scanned area. The margins of the resection site appeared smooth and regular, with no signs of local infiltration or postoperative complications. 

## Discussion

Etiology and epidemiology

SFTs are mesenchymal neoplasms with a fibroblastic origin. They affect mostly adult patients, between 50 and 60 years of age, and men and women at a similar rate. The incidence is estimated to be around one per million [5]. SFTs are characterized by the NGFI-A binding protein 2 and Signal Transducer and Activator of Transcription 6 (NAB2-STAT6) gene fusion, which is caused by recurrent intra-chromosomal paracentric inversion involving the long arm of chromosome 12 [9].

Clinical features

SFTs are usually asymptomatic, but when symptoms occur, they depend on the tumor's location. These manifestations can include pain, shortness of breath, weight loss, sudden rapid growth, and nasal obstruction [4]. SFTs are highly angiogenic tumors, which are visible on imaging and during surgery. This angiogenesis is present within the tumor itself, but also in large, tortuous extra-anatomical blood vessels surrounding it. These vessels are extremely friable, making the surgical procedure particularly hazardous for large tumors in confined anatomical spaces [10]. SFTs are characterized by the NAB2-STAT6 oncogenic fusion, which is pathognomonic for this tumor type, and this fusion is believed to drive the overexpression of vascular endothelial growth factor (VEGF), contributing to the highly vascular nature of SFTs [11]. The prominent vascularity is clinically relevant, as it may influence both tumor growth dynamics and the risk of intraoperative bleeding, emphasizing the importance of careful preoperative planning and vascular assessment in the management of these tumors. SFTs can sometimes present with clinical signs of hypoglycemia, which is caused by the secretion of IGFs [12].

In this patient, the neoplasm appeared in an unusual location. The presence of the tumor in a confined anatomical space like the area of the elbow joint can lead to clinical challenges, including difficulties in surgical access and the management of potential vascular complications. Furthermore, the patient experienced periodic numbness in the fingers, which is not specific and highlights how SFTs in the extremities can impact surrounding structures and present with functional abnormalities. This emphasizes the importance of considering SFTs in the differential diagnosis for masses in unusual locations, and in situations when patients present with symptoms affecting local function.

Diagnostic process

The process of diagnosis involves a combination of methods, but histopathologic and immunohistochemical examinations after excision are necessary to confirm the diagnosis of SFT [13]. Imaging techniques such as CT and magnetic resonance imaging (MRI) help reveal the proliferation of fibrous tissues and visualize the details of the tumor and adjacent tissue, aiding in decisions for surgical removal. However, CT and MRI features of SFTs are not specific, usually showing well-defined, lobulated masses of soft tissue that push aside nearby anatomical structures. These methods also help reveal the malignant features of the SFTs, including hemorrhages, infiltrating growths, necrosis, and lung metastasis [14]. When it comes to immunohistochemical staining, CD34, CD99, STAT6, and B-cell lymphoma 2 (Bcl-2) are useful positive immunohistochemical markers for SFT. Furthermore, identification of the NAB2-STAT6 fusion gene can also provide important information [15]. 

In our patient, histopathological examination confirmed the diagnosis of SFT, with the tumor exhibiting the characteristic features, including positive staining for CD34 and CD99. Furthermore, the CT angiography provided crucial information about the tumor’s blood supply and vascularization, which was essential in choosing the accurate surgical approach.

Differential diagnosis

Differential diagnosis of SFT is challenging due to its diverse histological patterns. Accurate diagnosis requires an integration of clinical presentation, histomorphology, immunohistochemistry, and molecular genetics. Key differential diagnoses include synovial sarcoma, malignant peripheral nerve sheath tumor, dedifferentiated liposarcoma, sarcomatoid mesothelioma, and other spindle cell neoplasms more common in specific anatomical locations [3]. Immunohistochemical markers such as CD34 and STAT6, along with detection of the NAB2-STAT6 fusion gene, are crucial in distinguishing SFT from these mimics [3,15]. Recognizing these features ensures appropriate diagnosis and management, particularly in extra-pleural sites where other soft tissue tumors predominate.

Treatment

The primary treatment for localized SFT is complete surgical resection [8]. Another treatment option is radiotherapy. It has been shown that resection and radiotherapy significantly lower the risk of local recurrence, as compared to oncologic resection alone [16]. The management of metastatic disease is challenging and requires decisions by a multidisciplinary team. The first line of treatment usually includes anthracyclines, with doxorubicin monotherapy being preferred. Additionally, metastasectomy or high-dose ablative radiotherapy could be valuable methods for managing metastatic disease. Patients with SFT need long-term follow-up due to the high risk of relapse even many years after the initial diagnosis [17].

In this case, the tumor was resected with a clear margin, which is essential to minimize the risk of recurrence. The presence of a highly vascularized tumor near the elbow joint posed significant surgical challenges, including the management of large blood vessels and the need to achieve complete tumor removal while preserving joint function. Initially, preoperative embolization was considered to reduce the tumor’s vascularity and size, thereby minimizing intraoperative bleeding. However, a detailed evaluation of CT angiography revealed that the feeding vessels were of adequate caliber and anatomical accessibility, allowing for their safe identification and ligation early in the surgical procedure. Although preoperative embolization has been shown to reduce blood loss in various highly vascular soft tissue tumors, it carries risks such as ischemic complications and prolonged operative time. This assessment led to the decision to proceed without embolization, optimizing surgical control while avoiding the risks associated with the endovascular procedure.

The long-term follow-up showed no pathological changes or recurrence, but it is necessary even after successful resection.

## Conclusions

This case highlights the importance of considering SFT in the differential diagnosis of slow-growing, vascularized soft tissue masses, even in rare locations like the elbow. Detailed preoperative imaging, including CT angiography, was crucial for surgical planning and allowed safe intraoperative ligation of feeding vessels, eliminating the need for embolization. Complete surgical excision resulted in favorable clinical outcomes. Given the potential for delayed recurrence or malignant transformation, long-term surveillance remains essential. Effective management of solitary fibrous tumors requires timely diagnosis, individualized surgical planning based on anatomical and histopathological characteristics, and coordinated care within a multidisciplinary team.

## References

[1] Klemperer P, Coleman BR (1992). Primary neoplasms of the pleura. A report of five cases. Am J Ind Med.

[2] Ros A, Cortés J, Belda T, Magán A, Illán-Gambín FJ, Aracil E, Serra C (2020). Fibrous solitary tumor, a rare and ubiquitous neoplasy. J Surg Case Rep.

[3] Tariq MU, Din NU, Abdul-Ghafar J, Park YK (2021). The many faces of solitary fibrous tumor; diversity of histological features, differential diagnosis and role of molecular studies and surrogate markers in avoiding misdiagnosis and predicting the behavior. Diagn Pathol.

[4] Martin-Broto J, Mondaza-Hernandez JL, Moura DS, Hindi N (2021). A comprehensive review on solitary fibrous tumor: new insights for new horizons. Cancers (Basel).

[5] Olson NJ, Linos K (2018). Dedifferentiated solitary fibrous tumor: a concise review. Arch Pathol Lab Med.

[6] Al-Shanawani BN, Al-Qattan MM, Arafah MM, Al-Motairi MI (2015). A solitary fibrous tumor of the upper limb. Saudi Med J.

[7] Toğral G, Tepedelenlioğlu HE, Akgün E, Korkmaz İ, Tolunay T (2024). The functional and clinical outcomes of primary and metastatic malignancies of the elbow. Jt Dis Relat Surg.

[8] Chandanwale SS, Gore CR, Sammi AB, Shah KR, Kaur PR (2014). Recurrent solitary fibrous tumor in distal lower extremity: An extremely rare entity. Int J Appl Basic Med Res.

[9] Demicco EG, Wagner MJ, Maki RG, Gupta V, Iofin I, Lazar AJ, Wang WL (2017). Risk assessment in solitary fibrous tumors: validation and refinement of a risk stratification model. Mod Pathol.

[10] Smrke A, Thway K, H Huang P, Jones RL, Hayes AJ (2021). Solitary fibrous tumor: molecular hallmarks and treatment for a rare sarcoma. Future Oncol.

[11] de Bernardi A, Dufresne A, Mishellany F, Blay JY, Ray-Coquard I, Brahmi M (2022). Novel therapeutic options for solitary fibrous tumor: antiangiogenic therapy and beyond. Cancers (Basel).

[12] Thway K, Ng W, Noujaim J, Jones RL, Fisher C (2016). The current status of solitary fibrous tumor: diagnostic features, variants, and genetics. Int J Surg Pathol.

[13] Jiang L, Hu C, Chen L, Chen L (2019). Diagnosis of solitary fibrous tumor in the submandibular gland: case report and literature review. J Clin Ultrasound.

[14] Zhanlong M, Haibin S, Xiangshan F, Jiacheng S, Yicheng N (2016). Variable solitary fibrous tumor locations: CT and MR imaging features. Medicine (Baltimore).

[15] Vogels RJ, Vlenterie M, Versleijen-Jonkers YM (2014). Solitary fibrous tumor - clinicopathologic, immunohistochemical and molecular analysis of 28 cases. Diagn Pathol.

[16] Haas RL, Walraven I, Lecointe-Artzner E (2020). Extrameningeal solitary fibrous tumors-surgery alone or surgery plus perioperative radiotherapy: a retrospective study from the global solitary fibrous tumor initiative in collaboration with the Sarcoma Patients EuroNet. Cancer.

[17] Janik AM, Terlecka A, Spałek MJ (2023). Diagnostics and treatment of extrameningeal solitary fibrous tumors. Cancers (Basel).

